# Supplementary material for: Retracing Phylogenetic, Host and Geographic Origins of Coronaviruses with Coloured Genomic Bootstrap Barcodes: SARS-CoV and SARS-CoV-2 as Case Studies
Source: Viruses. 2023 Jan 31;15(2):406. doi: 10.3390/v15020406 (PMC9961909; doi:10.3390/v15020406)
Supplement: Supplementary file 1 [file viruses-15-00406-s001.zip › viruses-2119850-supplementary.pdf]

Table S1: Origin of the 75 genomes of *Sarbecovirus* used in this study

Virus name	**Accession number**	**Host species**	**Geographic origin**	**Reference**
SARS-CoV HsRef*	NC_004718[1]	*Homo sapiens*	Canada	He et al. (2004)
SARS-CoV HsGD01*	AY278489[1]	*Homo sapiens*	China	Wu et al. (2003)
SARS-CoV HsZS-A*	AY394997[1]	*Homo sapiens*	China	Unpublished
SARS-CoV HsShanghaiQXC1*	AY463059[1]	*Homo sapiens*	China	Unpublished
SARS-CoV PlSZ3*	AY304486[1]	*Paguma larvata*	China	Guan et al. (2003)
SARS-CoV PlSZ61*	AY515512[1]	*Paguma larvata*	China	Unpublished
SARS-CoV PlGZ81*	AY545917[1]	*Paguma larvata*	China	Unpublished
As6526	KY417142[1]	*Aselliscus stoliczkanus*	Yunnan (China)	Hu et al. (2017)
RaLYRa11*	KF569996[1]	*Rhinolophus affinis*	Yunnan (China)	He et al. (2014)
RaYN2018A*	MK211375[1]	*Rhinolophus affinis*	Yunnan (China)	Han et al. (2019)
RaYN2018B*	MK211376[1]	*Rhinolophus affinis*	Yunnan (China)	Han et al. (2019)
RaYN2018C*	MK211377[1]	*Rhinolophus affinis*	Yunnan (China)	Han et al. (2019)
RaYN2018D*	MK211378[1]	*Rhinolophus affinis*	Yunnan (China)	Han et al. (2019)
Rf1	DQ412042[1]	*Rhinolophus ferrumequinum*[T1]	Hubei (China)	Li et al. (2005)
Rf4092	KY417145[1]	*Rhinolophus ferrumequinum*[T1]	Yunnan (China)	Hu et al. (2017)
RfJiyuan-84*	KY770860[1]	*Rhinolophus ferrumequinum*[T1]	Henan (China)	Lin et al. (2017)
RfV273*	DQ648856[1]	*Rhinolophus ferrumequinum*[T1]	Yunnan (China)	Tang et al. (2006)
RfYNLF/31C*	KP886808[1]	*Rhinolophus ferrumequinum*[T1]	Yunnan (China)	Lau et al. (2015)
RmYN07*	EPI_ISL_1699447[2]	*Rhinolophus malayanus*	Yunnan (China)	Zhou et al. (2021)
Rmac1*	DQ412043[1]	*Rhinolophus macrotis*[T2]	Hubei (China)	Li et al. (2005)
Rmac279*	DQ648857[1]	*Rhinolophus macrotis*[T2]	Yunnan (China)	Tang et al. (2006)
RmoLongquan140*	KF294457[1]	*Rhinolophus monoceros*[T3]	Zhejiang (China)	Lin et al. (2017)
RpF46*	KU973692[1]	*Rhinolophus pusillus*	Yunnan (China)	Wang et al. (2017)
RpShaanxi2011	JX993987[1]	*Rhinolophus pusillus*	Shaanxi (China)	Yang et al. (2013)
Rpe3	DQ071615[1]	*Rhinolophus pearsonii*	Guangxi (China)	Li et al. (2005)
Rs3367	KC881006[1]	*Rhinolophus sinicus*	Yunnan (China)	Ge et al. (2013)

Rs4081	KY417143[1]	*Rhinolophus sinicus*	Yunnan (China)	Hu et al. (2017)
Rs4084	KY417144[1]	*Rhinolophus sinicus*	Yunnan (China)	Hu et al. (2017)
Rs4231	KY417146[1]	*Rhinolophus sinicus*	Yunnan (China)	Hu et al. (2017)
Rs4237	KY417147[1]	*Rhinolophus sinicus*	Yunnan (China)	Hu et al. (2017)
Rs4247	KY417148[1]	*Rhinolophus sinicus*	Yunnan (China)	Hu et al. (2017)
Rs4255	KY417149[1]	*Rhinolophus sinicus*	Yunnan (China)	Hu et al. (2017)
Rs4874	KY417150[1]	*Rhinolophus sinicus*	Yunnan (China)	Hu et al. (2017)
Rs7327	KY417151[1]	*Rhinolophus sinicus*	Yunnan (China)	Hu et al. (2017)
Rs9401	KY417152[1]	*Rhinolophus sinicus*	Yunnan (China)	Hu et al. (2017)
RsAnlong103*	KY770858[1]	*Rhinolophus sinicus*	Guizhou (China)	Lin et al. (2017)
RsHKU3-1*	DQ022305[1]	*Rhinolophus sinicus*	Hong-Kong (China)	Lau et al. (2005)
RsHKU3-7*	GQ153542[1]	*Rhinolophus sinicus*	Hong-Kong (China)	Lau et al. (2010)
RsHKU3-12*	GQ153547[1]	*Rhinolophus sinicus*	Hong-Kong (China)	Lau et al. (2010)
RsHuB2013*	KJ473814[1]	*Rhinolophus sinicus*	Hubei (China)	Wu et al. (2016)
RsSHC014	KC881005[1]	*Rhinolophus sinicus*	Yunnan (China)	Ge et al. (2013)
RstYN03*	EPI_ISL_1699443[2]	*Rhinolophus stheno*[T4]	Yunnan (China)	Zhou et al. (2021)
RstYN09*	EPI_ISL_1699449[2]	*Rhinolophus stheno*[T4]	Yunnan (China)	Zhou et al. (2021)
RspSC2018*	MK211374[1]	*Rhinolophus* sp.	Sichuan (China)	Han et al. (2019)
SARS-CoV-2 HsRef*	NC_045512[1]	*Homo sapiens*	Hubei (China)	Wu et al. (2020)
SARS-CoV-2 HsAlpha*	OK581397[1]	*Homo sapiens*	USA	Unpublished
SARS-CoV-2 HsBeta*	OK238749[1]	*Homo sapiens*	USA	Unpublished
SARS-CoV-2 HsDelta*	OM793985[1]	*Homo sapiens*	USA	Unpublished
SARS-CoV-2 HsEpsilon*	OM432589[1]	*Homo sapiens*	USA	Unpublished
SARS-CoV-2 HsGamma*	OK630532[1]	*Homo sapiens*	USA	Unpublished
SARS-CoV-2 HsOmicron*	OP010674[1]	*Homo sapiens*	USA	Unpublished
SARS-CoV-2 NvCDC*	MW562276[1]	*Neovison vison*	USA	Unpublished
SARS-CoV-2 MlNB03*	MT457400[1]	*Mustela lutreola*	Netherlands	Unpublished
RaTG13	MN996532[1]	*Rhinolophus affinis*	Yunnan (China)	Zhou P. et al. (2020)

RacCS203	MW251308[1]	*Rhinolophus acuminatus*	Thailand	Wacharapluesadee et al. (2021)
RmYN02*	EPI_ISL_412977[2]	*Rhinolophus malayanus*	Yunnan (China)	Zhou H. et al. (2020)
RmBANAL52*	EPI_ISL_4302644[2]	*Rhinolophus malayanus*	Vientiane P. (Laos)	Temmam et al. (2022)
RmBANAL247*	EPI_ISL_4302648[2]	*Rhinolophus malayanus*	Vientiane P. (Laos)	Temmam et al. (2022)
RmarBANAL236*	EPI_ISL_4302647[2]	*Rhinolophus marshalli*	Vientiane P. (Laos)	Temmam et al. (2022)
RpBANAL103*	EPI_ISL_4302645[2]	*Rhinolophus pusillus*	Vientiane P. (Laos)	Temmam et al. (2022)
RpYN06*	EPI_ISL_1699446[2]	*Rhinolophus pusillus*	Yunnan (China)	Zhou et al. (2021)
RshSTT200	EPI_ISL_852605[2]	*Rhinolophus shameli*	Cambodia	Delaune et al. (2021)
MjGuangdong*	EPI_ISL_410721[2]	*Manis javanica*	Guangdong (China)	Xiao et al. (2020)
MjGuangxi*	EPI_ISL_410539[2]	*Manis javanica*	Guangxi (China)	Lam et al. (2020)
RsZXC21*	MG772934[1]	*Rhinolophus sinicus*	Zhejiang (China)	Hu et al. (2018)
RsZC45*	MG772933[1]	*Rhinolophus sinicus*	Zhejiang (China)	Hu et al. (2018)
RpPrC31*	EPI_ISL_1098866[2]	*Rhinolophus pusillus*	Yunnan (China)	Li et al. (2021)
RaTG15	GWHBAUP01000001[3]	*Rhinolophus affinis*	Yunnan (China)	Guo et al. (2021)
RstYN04*	EPI_ISL_1699444[2]	*Rhinolophus stheno*[T4]	Yunnan (China)	Zhou et al. (2021)
Rc-o319	LC556375[1]	*Rhinolophus cornutus*	Japan	Murakami et al. (2020)
RbBM48-31*	NC_014470[1]	*Rhinolophus blasii*	Bulgaria	Drexler et al. (2010)
RhGB01*	MW719567[1]	*Rhinolophus hipposideros*	United Kingdom	Crook et al. (2021)
RhKhosta1*	MZ190137[1]	*Rhinolophus hipposideros*	Russia	Alkhovsky et al. (2022)
RhKhosta2*	MZ190138[1]	*Rhinolophus hipposideros*	Russia	Alkhovsky et al. (2022)
RspKY72*	KY352407[1]	*Rhinolophus* sp.	Kenya	Tao and Tong (2019)

*original name slightly modified to be consistent with other names and to facilitate interpretations; 1: NCBI; 2: GISAID; 3: NGDC
Taxonomic issues (Burgin et al., 2020) = T1: currently *Rhinolophus nippon*; T2: currently *Rhinolophus episcopus*; T3 = the taxonomic assignation should be regarded as dubious because *Rhinolophus monoceros* is supposed to be endemic of Taiwan; T4: currently *Rhinolophus microglobosus*.

References

Alkhovsky, S., Lenshin, S., Romashin, A., Vishnevskaya, T., Vyshemirsky, O., Bulycheva, Y., Lvov, D., & Gitelman, A. (2022). SARS-like Coronaviruses in Horseshoe Bats (*Rhinolophus* spp.) in Russia, 2020. Viruses, 14(1), 113. https://doi.org/10.3390/v14010113

Drexler, J. F., Gloza-Rausch, F., Glende, J., Corman, V. M., Muth, D., Goettsche, M., Seebens, A., Niedrig, M., Pfefferle, S., Yordanov, S., Zhelyazkov, L., Hermanns, U., Vallo, P., Lukashev, A., Müller, M. A., Deng, H., Herrler, G., & Drosten, C. (2010). Genomic characterization of severe acute respiratory syndrome-related coronavirus in European bats and classification of coronaviruses based on partial RNA-dependent RNA polymerase gene sequences. Journal of virology, 84(21), 11336–11349. https://doi.org/10.1128/JVI.00650-10

Crook, J. M., Murphy, I., Carter, D. P., Pullan, S. T., Carroll, M., Vipond, R., Cunningham, A. A., & Bell, D. (2021). Metagenomic identification of a new sarbecovirus from horseshoe bats in Europe. Scientific reports, 11(1), 14723. https://doi.org/10.1038/s41598-021-94011-z

Ge, X. Y., Li, J. L., Yang, X. L., Chmura, A. A., Zhu, G., Epstein, J. H., Mazet, J. K., Hu, B., Zhang, W., Peng, C., Zhang, Y. J., Luo, C. M., Tan, B., Wang, N., Zhu, Y., Crameri, G., Zhang, S. Y., Wang, L. F., Daszak, P., & Shi, Z. L. (2013). Isolation and characterization of a bat SARS-like coronavirus that uses the ACE2 receptor. Nature, 503(7477), 535–538. https://doi.org/10.1038/nature12711

Guan, Y., Zheng, B. J., He, Y. Q., Liu, X. L., Zhuang, Z. X., Cheung, C. L., Luo, S. W., Li, P. H., Zhang, L. J., Guan, Y. J., Butt, K. M., Wong, K. L., Chan, K. W., Lim, W., Shortridge, K. F., Yuen, K. Y., Peiris, J. S., & Poon, L. L. (2003). Isolation and characterization of viruses related to the SARS coronavirus from animals in southern China. Science (New York, N.Y.), 302(5643), 276–278. https://doi.org/10.1126/science.1087139

Guo, H., Hu, B., Si, H. R., Zhu, Y., Zhang, W., Li, B., Li, A., Geng, R., Lin, H. F., Yang, X. L., Zhou, P., & Shi, Z. L. (2021). Identification of a novel lineage bat SARS-related coronaviruses that use bat ACE2 receptor. Emerging microbes & infections, 10(1), 1507–1514. https://doi.org/10.1080/22221751.2021.1956373

Han, Y., Du, J., Su, H., Zhang, J., Zhu, G., Zhang, S., Wu, Z., & Jin, Q. (2019). Identification of Diverse Bat Alphacoronaviruses and Betacoronaviruses in China Provides New Insights Into the Evolution and Origin of Coronavirus-Related Diseases. Frontiers in microbiology, 10, 1900. https://doi.org/10.3389/fmicb.2019.01900

He, B., Zhang, Y., Xu, L., Yang, W., Yang, F., Feng, Y., Xia, L., Zhou, J., Zhen, W., Feng, Y., Guo, H., Zhang, H., & Tu, C. (2014). Identification of diverse alphacoronaviruses and genomic characterization of a novel severe acute respiratory syndrome-like coronavirus from bats in China. Journal of virology, 88(12), 7070–7082. https://doi.org/10.1128/JVI.00631-14

He, R., Dobie, F., Ballantine, M., Leeson, A., Li, Y., Bastien, N., Cutts, T., Andonov, A., Cao, J., Booth, T. F., Plummer, F. A., Tyler, S., Baker, L., & Li, X. (2004). Analysis of multimerization of the SARS coronavirus nucleocapsid protein. Biochemical and biophysical research communications, 316(2), 476–483. https://doi.org/10.1016/j.bbrc.2004.02.074

Hu, B., Zeng, L. P., Yang, X. L., Ge, X. Y., Zhang, W., Li, B., Xie, J. Z., Shen, X. R., Zhang, Y. Z., Wang, N., Luo, D. S., Zheng, X. S., Wang, M. N., Daszak, P., Wang, L. F., Cui, J., & Shi, Z. L. (2017). Discovery of a rich gene pool of bat SARS-related coronaviruses provides new insights into the origin of SARS coronavirus. PLoS pathogens, 13(11), e1006698. https://doi.org/10.1371/journal.ppat.1006698

Hu, D., Zhu, C., Ai, L., He, T., Wang, Y., Ye, F., Yang, L., Ding, C., Zhu, X., Lv, R., Zhu, J., Hassan, B., Feng, Y., Tan, W., & Wang, C. (2018). Genomic characterization and infectivity of a novel SARS-like coronavirus in Chinese bats. Emerging microbes & infections, 7(1), 154. https://doi.org/10.1038/s41426-018-0155-5

Lam, T. T., Jia, N., Zhang, Y. W., Shum, M. H., Jiang, J. F., Zhu, H. C., Tong, Y. G., Shi, Y. X., Ni, X. B., Liao, Y. S., Li, W. J., Jiang, B. G., Wei, W., Yuan, T. T., Zheng, K., Cui, X. M., Li, J., Pei, G. Q., Qiang, X., Cheung, W. Y., … Cao, W. C. (2020). Identifying SARS-CoV-2-related coronaviruses in Malayan pangolins. Nature, 583(7815), 282–285. https://doi.org/10.1038/s41586-020-2169-0

Lau, S. K., Feng, Y., Chen, H., Luk, H. K., Yang, W. H., Li, K. S., Zhang, Y. Z., Huang, Y., Song, Z. Z., Chow, W. N., Fan, R. Y., Ahmed, S. S., Yeung, H. C., Lam, C. S., Cai, J. P., Wong, S. S., Chan, J. F., Yuen, K. Y., Zhang, H. L., & Woo, P. C. (2015). Severe Acute Respiratory Syndrome (SARS) Coronavirus ORF8 Protein Is Acquired from SARS-Related Coronavirus from Greater Horseshoe Bats through Recombination. Journal of virology, 89(20), 10532–10547. https://doi.org/10.1128/JVI.01048-15

Lau, S. K., Li, K. S., Huang, Y., Shek, C. T., Tse, H., Wang, M., Choi, G. K., Xu, H., Lam, C. S., Guo, R., Chan, K. H., Zheng, B. J., Woo, P. C., & Yuen, K. Y. (2010). Ecoepidemiology and complete genome comparison of different strains of severe acute respiratory syndrome-related Rhinolophus bat coronavirus in China reveal bats as a reservoir for acute, self-limiting infection that allows recombination events. Journal of virology, 84(6), 2808–2819. https://doi.org/10.1128/JVI.02219-09

Lau, S. K., Woo, P. C., Li, K. S., Huang, Y., Tsoi, H. W., Wong, B. H., Wong, S. S., Leung, S. Y., Chan, K. H., & Yuen, K. Y. (2005). Severe acute respiratory syndrome coronavirus-like virus in Chinese horseshoe bats. Proceedings of the National Academy of Sciences of the United States of America, 102(39), 14040–14045. https://doi.org/10.1073/pnas.0506735102

Li, W., Shi, Z., Yu, M., Ren, W., Smith, C., Epstein, J. H., Wang, H., Crameri, G., Hu, Z., Zhang, H., Zhang, J., McEachern, J., Field, H., Daszak, P., Eaton, B. T., Zhang, S., & Wang, L. F. (2005). Bats are natural reservoirs of SARS-like coronaviruses. Science (New York), 310(5748), 676–679. https://doi.org/10.1126/science.1118391

Lin, X. D., Wang, W., Hao, Z. Y., Wang, Z. X., Guo, W. P., Guan, X. Q., Wang, M. R., Wang, H. W., Zhou, R. H., Li, M. H., Tang, G. P., Wu, J., Holmes, E. C., & Zhang, Y. Z. (2017). Extensive diversity of coronaviruses in bats from China. Virology, 507, 1–10. https://doi.org/10.1016/j.virol.2017.03.019

Murakami, S., Kitamura, T., Suzuki, J., Sato, R., Aoi, T., Fujii, M., Matsugo, H., Kamiki, H., Ishida, H., Takenaka-Uema, A., Shimojima, M., & Horimoto, T. (2020). Detection and Characterization of Bat Sarbecovirus Phylogenetically Related to SARS-CoV-2, Japan. Emerging infectious diseases, 26(12), 3025–3029. https://doi.org/10.3201/eid2612.203386

Tang, X. C., Zhang, J. X., Zhang, S. Y., Wang, P., Fan, X. H., Li, L. F., Li, G., Dong, B. Q., Liu, W., Cheung, C. L., Xu, K. M., Song, W. J., Vijaykrishna, D., Poon, L. L., Peiris, J. S., Smith, G. J., Chen, H., & Guan, Y. (2006). Prevalence and genetic diversity of coronaviruses in bats from China. Journal of virology, 80(15), 7481–7490. https://doi.org/10.1128/JVI.00697-06

Tao, Y., & Tong, S. (2019). Complete Genome Sequence of a Severe Acute Respiratory Syndrome-Related Coronavirus from Kenyan Bats. Microbiology resource announcements, 8(28), e00548-19. https://doi.org/10.1128/MRA.00548-19

Temmam, S., Vongphayloth, K., Baquero, E., Munier, S., Bonomi, M., Regnault, B., Douangboubpha, B., Karami, Y., Chrétien, D., Sanamxay, D., Xayaphet, V., Paphaphanh, P., Lacoste, V., Somlor, S., Lakeomany, K., Phommavanh, N., Pérot, P., Dehan, O., Amara, F., Donati, F., … Eloit, M. (2022). Bat coronaviruses related to SARS-CoV-2 and infectious for human cells. Nature, 604(7905), 330–336. https://doi.org/10.1038/s41586-022-04532-4

Wacharapluesadee, S., Tan, C. W., Maneeorn, P., Duengkae, P., Zhu, F., Joyjinda, Y., Kaewpom, T., Chia, W. N., Ampoot, W., Lim, B. L., Worachotsueptrakun, K., Chen, V. C., Sirichan, N., Ruchisrisarod, C., Rodpan, A., Noradechanon, K., Phaichana, T., Jantarat, N., Thongnumchaima, B., Tu, C., … Wang, L. F. (2021). Evidence for SARS-CoV-2 related coronaviruses circulating in bats and pangolins in Southeast Asia. Nature communications, 12(1), 972. https://doi.org/10.1038/s41467-021-21240-1

Wang, L., Fu, S., Cao, Y., Zhang, H., Feng, Y., Yang, W., Nie, K., Ma, X., & Liang, G. (2017). Discovery and genetic analysis of novel coronaviruses in least horseshoe bats in southwestern China. Emerging microbes & infections, 6(3), e14. https://doi.org/10.1038/emi.2016.140

Wu, F., Zhao, S., Yu, B., Chen, Y. M., Wang, W., Song, Z. G., Hu, Y., Tao, Z. W., Tian, J. H., Pei, Y. Y., Yuan, M. L., Zhang, Y. L., Dai, F. H., Liu, Y., Wang, Q. M., Zheng, J. J., Xu, L., Holmes, E. C., & Zhang, Y. Z. (2020). A new coronavirus associated with human respiratory disease in China. Nature, 579(7798), 265–269. https://doi.org/10.1038/s41586-020-2008-3

Wu, Q., Zhang, Y., Lü, H., Wang, J., He, X., Liu, Y., Ye, C., Lin, W., Hu, J., Ji, J., Xu, J., Ye, J., Hu, Y., Chen, W., Li, S., Wang, J., Wang, J., Bi, S., & Yang, H. (2003). The E protein is a multifunctional membrane protein of SARS-CoV. Genomics, proteomics & bioinformatics, 1(2), 131–144. https://doi.org/10.1016/s1672-0229(03)01017-9

Wu, Z., Yang, L., Ren, X., Zhang, J., Yang, F., Zhang, S., & Jin, Q. (2016). ORF8-Related Genetic Evidence for Chinese Horseshoe Bats as the Source of Human Severe Acute Respiratory Syndrome Coronavirus. The Journal of infectious diseases, 213(4), 579–583. https://doi.org/10.1093/infdis/jiv476

Xiao, K., Zhai, J., Feng, Y., Zhou, N., Zhang, X., Zou, J. J., Li, N., Guo, Y., Li, X., Shen, X., Zhang, Z., Shu, F., Huang, W., Li, Y., Zhang, Z., Chen, R. A., Wu, Y. J., Peng, S. M., Huang, M., Xie, W. J., … Shen, Y. (2020). Isolation of SARS-CoV-2-related coronavirus from Malayan pangolins. Nature, 583(7815), 286–289. https://doi.org/10.1038/s41586-020-2313-x

Yang, L., Wu, Z., Ren, X., Yang, F., He, G., Zhang, J., Dong, J., Sun, L., Zhu, Y., Du, J., Zhang, S., & Jin, Q. (2013). Novel SARS-like betacoronaviruses in bats, China, 2011. Emerging infectious diseases, 19(6), 989–991. https://doi.org/10.3201/eid1906.121648

Zhou, H., Chen, X., Hu, T., Li, J., Song, H., Liu, Y., Wang, P., Liu, D., Yang, J., Holmes, E. C., Hughes, A. C., Bi, Y., & Shi, W. (2020). A Novel Bat Coronavirus Closely Related to SARS-CoV-2 Contains Natural Insertions at the S1/S2 Cleavage Site of the Spike Protein. Current biology : CB, 30(11), 2196–2203.e3. https://doi.org/10.1016/j.cub.2020.05.023

Zhou, H., Ji, J., Chen, X., Bi, Y., Li, J., Wang, Q., Hu, T., Song, H., Zhao, R., Chen, Y., Cui, M., Zhang, Y., Hughes, A. C., Holmes, E. C., & Shi, W. (2021). Identification of novel bat coronaviruses sheds light on the evolutionary origins of SARS-CoV-2 and related viruses. Cell, S0092-8674(21)00709-1. https://doi.org/10.1016/j.cell.2021.06.008

Zhou, P., Yang, X. L., Wang, X. G., Hu, B., Zhang, L., Zhang, W., Si, H. R., Zhu, Y., Li, B., Huang, C. L., Chen, H. D., Chen, J., Luo, Y., Guo, H., Jiang, R. D., Liu, M. Q., Chen, Y., Shen, X. R., Wang, X., Zheng, X. S., … Shi, Z. L. (2020). A pneumonia outbreak associated with a new coronavirus of probable bat origin. Nature, 579(7798), 270–273. https://doi.org/10.1038/s41586-020-2012-7
